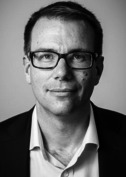# Assessing Methodologies to Synthesize α‐Sulfenylated Carbonyl Compounds by Green Chemistry Metrics

**DOI:** 10.1002/cssc.202002409

**Published:** 2020-11-23

**Authors:** Jèssica Margalef, Joseph S. M. Samec

**Affiliations:** ^1^ Departament de Química Física i Inorgànica Universitat Rovira i Virgili C/ Marcel lí Domingo, 1 43007 Tarragona Spain; ^2^ Department of Organic Chemistry Stockholm University Svante Arrhenius väg 16 C 106 91 Stockholm Sweden

**Keywords:** atom economy, carbonyl compounds, E factors, green metrics, sustainable chemistry

## Abstract

α‐Sulfenylated carbonyl compounds are important both as active pharmaceutical ingredients and as intermediates in organic synthesis. Owing to their relevance in synthetic organic chemistry, this Minireview focuses on assessing the most relevant synthetic procedures based on green chemistry metrics. The Minireview starts with the traditional routes and then focuses on more recently developed methodologies. These routes include sulfenylating reagents using organocatalysis, cross‐dehydrogenative couplings using in situ halogenations to prevent reactive intermediates in high concentrations, oxidative couplings using terminal oxidants such as DDQ or TEMPO, and redox‐neutral couplings using transition metal catalysis. These methodologies have been evaluated on the basis of atom economy, E factor, and the safety and toxicity of the transformations and the solvents used. Besides using green metrics to evaluate these novel methodologies, the synthetic utility is also assessed with regard to the availability of starting materials and the generality of the reactions. This Minireview aims to inspire researchers to apply green assessments to other methodologies and also for them to take measures to increase the greenness of their developed transformations.

## Graphical abstract

**How green was my method**? In this Minireview, the available routes towards α‐sulfenylated carbonyl compounds are assessed by means of green chemistry. The uses of both atom economy and E‐factor, as well as analysis of the safety and the nature of solvents, are necessary to estimate the greenness of each procedure. The greenness of many such established methodologies can be improved with only small adjustments.

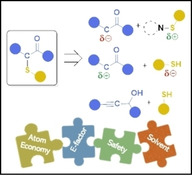

## 1 Introduction

**Figure 1:**
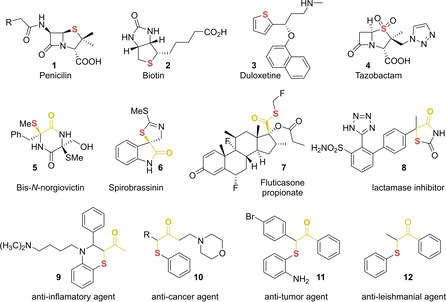
Selected natural products, drugs, and bioactive compounds bearing a sulfur atom.

Sulfur is present in many pharmaceuticals and other biologically active compounds (Figure 1).^[1]^ Interestingly, approximately 20 % of the approved FDA drugs contain sulfur atoms.^[2]^ Due to the wide applications of organosulfur compounds in organic and biological chemistry, the formation of new C−S bonds is an important topic. The synthesis of α‐sulfenylated carbonyl compounds is interesting since they can be derivatized to a variety of organosulfur compounds. For example, they provide easy access to S‐containing heterocycles,^[3]^ β‐keto sulfones^[4]^ and vicinal thioether alcohols.^[5]^ In addition, several biologically active compounds present a sulfenyl moiety in the α‐position of a carbonyl group (compounds **5**–**12**, Figure 1).[^1^, ^6^] Moreover, the sp^3^‐hybridized C−S bond in thioethers can be activated towards other functional groups if desired. For example, α‐sulfenylated carbonyl compounds can participate in cross‐coupling reactions^[7]^ and be transformed into olefins,^[8]^ organometallics,^[9]^ and halides.^[10]^ In the case of chiral α‐sulfenylated compounds, it has also been shown that the thioether moiety can also be transformed to other functional groups in an enantiospecific fashion.[^10^, ^11^]

**Scheme 1:**
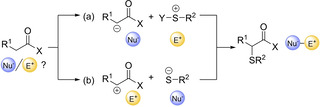
Two general approaches used for preparing α‐sulfenylated carbonyl compounds, taking into account the reactivity nature of the carbonyl compound.

From a retrosynthetic point of view, there are two approaches to prepare α‐sulfenylated carbonyl compounds from carbonyl derivatives (Scheme 1). The α‐position of a carbonyl functionality is usually considered a nucleophile, through prior formation of the enol/enolate intermediate, and therefore can react with an electrophilic sulfur reagent (Scheme 1a). However, the carbonyl can also act as an electrophile if it is properly activated, followed by reaction with thiolates (Scheme 1b).

The first approach, i. e. when the carbonyl acts as nucleophile, has been achieved with several electrophilic sulfur reagents, such as disulfides, *N*‐(phenylsulfanyl)succinimides/phthalimides/1,2,4‐triazoles, or sulfenyl chlorides.[^12^, ^13^, ^14^, ^15^, ^16^, ^17^] For the second approach, i. e. when the carbonyl acts as electrophile, there are two possibilities concerning the activation of the carbonyl substrates: i) a preformed α‐halogenated carbonyl compound can be used, which will undergo nucleophilic substitution with thiols^[18]^ or disulfides;^[19]^ ii) the substrate can be activated with the use of halogenated reagents and/or strong oxidants.^[20]^ Although there are several excellent reports based on both methodologies for the synthesis of α‐sulfenylated compounds, the required reagents involved in the reaction and the generation of large amounts of chemical waste are not desired. Therefore, the development of greener processes is desired. It is interesting to note that for both approaches, most of the methods used are not metal‐catalyzed processes, which is probably because of the inactivation of metal‐catalysts due to the nucleophilicity of the sulfur atom.^[21]^

In this mini review we analyze the methodologies developed since 2005 for the synthesis of α‐sulfenylated carbonyl compounds by means of green chemistry metrics. Green chemistry was introduced by Prof. Anastas and Prof. Warner in 1998 as an innovative approach to assess chemical reactions based on 12 principles.^[22]^ These principles concern waste generation of transformations, toxicity of reagents, intermediates and products, energy consumption and renewability and recyclability. Green chemistry has become a major topic that today influences all chemistry disciplines and has been widely accepted by both academia and industry. To evaluate green chemistry aspects of transformations is not trivial, especially academic reports where optimizations are mostly focused on yields and reaction times. In this Minireview we have assessed the green chemistry aspects of preparation of α‐sulfenylated carbonyl compounds using four criteria: 1) Atom economy; 2) E factor; 3) safety; and 4) solvents.

The atom economy metric was introduced by Prof. Trost in 1991 and is considered as the first green metric for chemical reactions.^[23]^ The atom economy is an easily applied metric used to determine the greenness of a reaction and is easily calculated by Equation 1:

(1) $$\mathrm{Atom}\;\mathrm{economy}=[M{}_{W}\left(\mathrm{product}\right)/\sum M{}_{W}\left(\mathrm{reagents}\right)]\times 100$$

However, as with all metrics there are shortcomings. For example, the atom economy does not consider yields, consumption of solvents or purification agents. The environmental factor (E factor),^[24]^ that was introduced by Prof. Sheldon takes into account all waste generated during work‐up and purification according to Equation 2:

(2) Efactor=massofallwaste/massofproduct

The E factor gives more information about the greenness of a chemical reaction as compared to atom economy, however it also has shortcomings such as not accounting for energy consumption^[25]^ and further external processing of waste generated during a process. Whereas the data to access atom economy is readily available even from scientific publications, the same is not true for the E factor. Thus, to assess the E factor for a reaction where all the data has not been given is difficult to achieve with high accuracy. However, factors that will affect the E factor can be estimated such as: yield of desired product, number of reaction steps from readily available starting materials, need for derivatization‐steps, and use of different reaction media and possibility to recycle catalyst.

Another term introduced by Prof. Sheldon is the Environmental quotient to estimate the toxicity and environmental unfriendliness of a waste. As there are no scales and no efficient way to calculate a value, this metric has not been widely used. However, toxicity and hazardous reagents, especially solvents, have gained attention and has become an independent research field.^[26]^ The toxicity and safety of reagents and solvents are central to green chemistry and today there are readily available charts of desired and less desired solvents widely applied in industry.^[27]^

In this review our ambition has been to assess the green metrics of different synthetic routes to α‐sulfenylated carbonyl compounds. We have used the following metrics.

Atom economy as it is widely accepted and also easily calculated. We have used the model substrate of each reported method to calculate the atom economy and estimate Atom economies below 45 % as poor, between 45–65 % as moderate, and above 65 % as good. The atom economy is only calculated on the α‐sulfenylation reaction and does not account for the transformation from readily accessible starting materials.

As it is not possible to accurately calculate E factors from scientific publications where e. g. column chromatography is used, we have instead included a discussion of what factors that affect the E factor for each methodology accessing the transformation from readily available starting materials to final products. What is most important for E factors are the overall yield of the final product, number of purifications, and change of solvents which usually are reflected by the number of reaction steps that require work‐up and purification. Other factors that affect the E factor are recyclability of catalysts, reagents and solvents. Thus, activation of substrates or derivatization steps in a synthetic route lower the E factor, whereas tandem reactions and strategies to recover solvents, reagents and catalysts are beneficial. Almost all academic studies use column chromatography to purify products. For several reasons including labor intensity, but also vast amounts of solvents and silica used that can be related to large E factors, column chromatography is avoided in industry. For this reason, we will not assess the work‐up or purification methodologies, and instead only take into account number of steps that require a purification. Although the E factor is not currently used to assess the energy consumption for producing a compound, there is a correlation between energy consumption and the E factor. All reagents that don't end up in the product as well as the solvents used, will need to be recycled or disposed and this will require energy input.

Safety includes both using highly reactive or toxic chemicals as well as harsh reaction conditions such as reactions under pressure. This also includes intermediates in a synthetic route. Moreover, highly exothermic reactions are considered adverse with respect to safety. Another factor that is important in industry is measured exotherms that can occur above the desired reaction. These are always carefully assessed in industry, however very rarely considered in academic studies. Thus, this has unfortunately not been taken into account in this Minireview. The choice of solvents has not been considered in the safety assessment.

An assessment of solvents in the reactions has been included. Unfortunately, many academic procedures still use halogenated solvents even though these solvents have restricted usage in industry. As the purifications use column chromatography and we propose that an up‐scaled process would not, all solvents in the work‐ups and purifications have been omitted in this assessment.

Even though we are aware that we are pointing to specific methodologies in this assessment and judging them, our main goal has not been to evaluate how good a synthetic route is. Instead, we compiled the methodologies that we found and assessed them on the basis described above. Many of the methodologies would with small adjustments become much greener than what has been reported. We hope that this Minireview will not be used as a judgement of previous reports, but more to inspire and encourage researchers to take measures already during the development of novel methodologies to increase the greenness.

## 2 Methods for the Synthesis of α‐Sulfenylated Carbonyl Compounds

The Minireview will assess novel methodologies based on the metrics described above. The methodologies are grouped into:

- Electrophilic sulfenylations
- Cross‐dehydrogenative couplings
- Oxidative couplings
- Redox neutral couplings

### 2.1 Electrophilic sulfenylations

Among the methods based on the use of nucleophilic carbonyl compounds, organocatalytic electrophilic sulfenylations are the most widely employed, especially for preparing chiral sulfenylated compounds.[^12^, ^13^, ^14^, ^15^, ^16^, ^17^] The most common organocatalysts used are chiral molecules that are readily available from the chiral pool, such as L‐prolinol derivatives or cinchonidine and quinine alkaloid derivatives. Therefore, although some examples of metal‐catalyzed α‐sulfenylations have been reported,^[21]^ metal‐free processes are the most common. Regarding the substrate scope, several activated carbonyl compounds such as oxindoles,^[13]^ β‐ketoesters^[14]^ and azalactones,^[15]^ among others^[17]^ can be efficiently sulfenylated. However, fewer methodologies exist for other less reactive substrates, such as ketones or aldehydes,^[16]^ which limits the scope of the reaction.

**Scheme 2:**
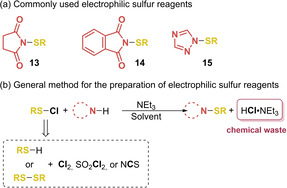
Commonly used electrophilic sulfur reagents (a) and general method for their preparation (b).

Another important limitation is that the process relies on the use of electrophilic sulfur reagents, which are not ideal in terms of green chemistry. The most typical are *N*‐(benzyl/aryl)succinimide, ‐phthalimide or −1,2,4‐triazole (**13**–**15**, Scheme 2). These sulfur reagents are generally prepared from the desired chlorinated thiol and nitrogen‐containing leaving group, in presence of triethyl amine (Scheme 2).[^16a^, ^28^] Sulfenyl chloride derivatives are usually prepared through chlorination of the corresponding thiol or disulfide and chlorine gas, sulfuryl chloride or *N*‐chlorosuccinimide, and used without further purification.^[29]^ This implies two extra derivatization steps for their preparation, which results in the generation of high amounts of halogen‐containing waste while increasing the amount of solvent used in the overall process. In some reported procedures the purification of the sulfenylated reagents is performed by column chromatography.^[30]^ To minimize the amount of organic solvents, the purification of the resulting sulfenylated reagents through column chromatography should be avoided. In most of the cases it is possible to perform a recrystallization, since succinimide and phthalimide compounds **13** and **14** are solid.^[31]^ For example, the synthesis of *N*‐(benzenesulfenyl)phthalimide (**14**, R=Bn) can be carried out from benzenesulfenyl chloride and phthalimide in the presence of triethyl amine and obtained in its pure form without performing column chromatography. The formed triethylamine hydrochloride salts can be removed through filtration and the resulting crude product can be recrystallized from ethanol.^[28b]^ In this particular case, the E factor of the preparation of **14** from the freshly prepared benzenemethanesulfenyl chloride and commercially available *N*‐chlorophthalimide will be quite low,^[32]^ whereas if the purification of the sulfenylated reagents is performed by column chromatography, this value would considerably increase. However, the atom economy of this step is 50 %, thus the total atom economy of the whole process will considerably decrease, reflecting the high amount of chemical waste generated, which is even magnified when considering that during the sulfenylation process a nitrogen nucleofuge is generated.

**Scheme 3:**
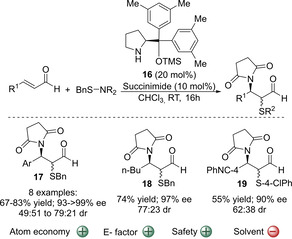
Catalytic asymmetric aminosulfenylation of α,β‐unsaturated aldehydes catalyzed by organocatalyst **16**.

Nevertheless, the organocatalytic α‐sulfenylation is a powerful approach towards chiral α‐sulfenylated carbonyl compounds, and to date several catalytic systems have been reported that allowed their preparation.[^12^, ^13^, ^14^, ^15^, ^16^, ^17^] Therefore, the development of a more sustainable version of this reaction is desired. In this respect the methodology developed by Córdova and co‐workers is noteworthy. They realized that sulfur electrophiles (Nu−E) include a masked nucleophilic component and therefore, after the electrophilic sulfenylation occurs, a domino reaction mediated by the generated nucleofuge would be possible, becoming a waste‐free transformation. This opens the possibility of preparing even more functionalized sulfenylated carbonyl compounds in only one step, thus improving the atom economy of the reaction and minimizing the amount of consumed solvents and produced waste. The authors reported for the first time the organocatalytic asymmetric aminosulfenylation reaction of commercially available cinnamic aldehydes and *N*‐(benzylthio)succinimide to yield a range of *syn*‐ and *anti‐*β‐amino‐α‐mercaptoaldehydes **17**–**18** in good yields (60–83 %) and high enantioselectivities (93 to >99 % *ee*; Scheme 3).^[16a]^ Although the diastereomeric ratio was poor (typically c.a. 50 : 50), in most of the cases pure s*yn*‐ and *anti*‐products could be obtained through column chromatography. The catalytic system also tolerated a succinimide reagent bearing a *p*‐chlorophenylsulfenyl group, although the yield was lower in this case (Scheme 3). This system offers a greener approach towards valuable compounds with a β‐amino‐α‐mercaptocarbonyl motif^[33]^ in high atom economy (89 %), if only the sulfenylation reaction is considered. Due to the use of *N*‐(benzylthio)succinimide, the E factor gets slightly higher when considering the overall process. Nevertheless, the cinnamic aldehyde derivatives used are generally available, and the chiral organocatalyst used (**16**) can be prepared through alcohol protection of commercially available α,α‐diphenyl‐2‐pyrrolidinemethanol in only one step. Therefore, although the process is not completely waste‐free, it considerably reduces the amount of chemical waste of the overall transformation compared with traditional methods. It should be noted that although chloroform is used as a solvent, the reaction can be also performed in toluene, which is preferred over chloroform, with similar enantioselectivities (95 % vs. >99 % *ee*) and diastereoselectivities (52 : 48 d.r.), although a lower yield was recorded (49 % yield).

**Scheme 4:**
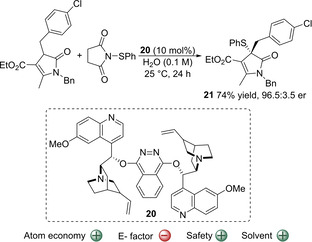
Catalytic enantioselective α‐sulfenylation of a deconjugated butyrolactam in water catalyzed by cinchona alkaloid **20**.

Another methodology that presents an advantage compared to classical sulfenylation protocols is the α‐sulfenylation of deconjugated butyrolactams catalyzed by a dimeric cinchona alkaloid **20**.^[17d]^ The authors found that this reaction could be accelerated when water was present in the reaction media. Therefore, the optimal solvent media was a mixture of *t*BuPh/H_2_O (1 : 9). The reaction delivered a range of highly substituted and densely functionalized γ‐lactams, bearing a quaternary stereogenic center, in moderate to high yields and generally with high enantioselectivities. The rate acceleration observed in water‐enriched media was attributed possibly to the hydrophobic hydration effect. Interestingly, the authors showed that the reaction could be also performed at 1 mmol scale in full aqueous media, yielding compound **21** in similar yields and enantioselectivities than in *t*BuPh/H_2_O (1 : 9) mixture (Scheme 4; 74 % yield and 96.5 : 3.5 e.r.).^[34]^ Therefore, it constitutes a rare and good example of synthesizing enantiomerically pure compounds while completely avoiding the use of undesired organic solvents, which is promising. In addition, it offers the possibility of recovering the catalysts by extraction if a water‐soluble catalyst is used. This practice would lower the E factor of the process. Unfortunately, catalyst **20** is likely not soluble in water, which hampers its recycling. The crude sulfenylated product is extracted using EtOAc and the product must be purified by column chromatography, followed by a recrystallization. If catalyst **20** was water‐soluble, it might be possible to avoid the column chromatography, which would make the E factor more favorable. Other features of the process that increase the E factor are the use of *N*‐(phenylsulfanyl)succinimide, the deconjugated butyrolactams are prepared in 4 steps (involving three column chromatography) and the ligand is prepared in one step from commercially available quinidine and 1,4‐dichlorophthalazin in presence of 3 equivalents of K_2_CO_3_ (involving a column chromatography). Despite the high estimated E factor for these processes, the atom economy is 71 % which is a good value for this class of reactions. Finally, the fact that the reaction can be performed in the presence of water means that the reaction is not moisture sensitive, so no complicated Schlenk techniques are needed and this is preferred for industrial applications.

**Scheme 5:**
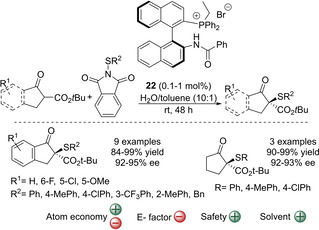
Asymmetric sulfenylation of β‐ketoesters catalyzed by quaternary phosphonium bromide **22**.

The α‐sulfenylation of β‐ketoesters developed by Maruoka and co‐workers can be also performed in the presence of water. In this case, the reaction is performed in a mixture of water and toluene in a 10 : 1 ratio and uses a bifunctional quaternary phosphonium bromide possessing an amide moiety (**22**, Scheme 5).^[14c]^ The tuning of the amide moiety was crucial to achieve high yields (84–99 %) and enantioselectivities (92–95 % *ee*). The reaction tolerated several substituents on the aryl ring of the 1‐oxo‐2‐indanecarboxylates as well as different substituents on the *N*‐sulfenyl group. Importantly, the catalyst loading was as low as 0.1 mol%, which demonstrates the practicality of the reaction (usually 10–20 mol% of catalyst loading is used). The α‐sulfenylation reaction of 2‐oxocyclopentanecarboxylate, also proceeded with high catalytic performance (90–99 % yield, 92–93 % *ee*), albeit in this case 1 mol% of chiral catalyst was used. The reaction gives a moderate atom economy (63 %). In contrast to the previous methodologies (see above) and since catalyst **22** is water‐soluble, and thus recyclable. However, as far as we know it was not shown by the authors. The possibility of performing the reaction in full aqueous media was not shown either. Unfortunately, the amount of solvent used during the whole process is quite high due to ligand **22**, β‐ketoesters and phthalimide sulfenylating agents are not commercially available so three further synthetic steps must be carried out, two of them involving further column chromatography. Moreover, EtOAc was used to extract the crude product, which was further purified by column chromatography. Despite the high estimated E factor for this process, we believe that this reaction presents several interesting features, such as low catalyst loadings and the use of a water‐soluble catalyst. These features could be further exploited to achieve a better E factor.

### 2.2 Cross‐dehydrogenative couplings

**Scheme 6:**
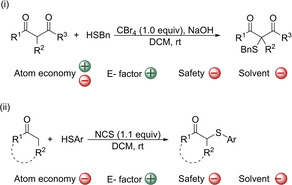
First examples of the oxidative coupling between thiols and carbonyl compounds. (i) Thiolation of several β‐dicarbonyl compounds promoted by CBr_4_, and (ii) thiolation of cyclic and linear ketones promoted by NCS.

The strategy of activating the carbonyl compound to act as an electrophile offers the possibility of avoiding the use of electrophilic sulfur reagents. In contrast, thiols can be used as thiolating agents, which are commercially available and allow more variability of the sulfenyl group introduced. Of course, the use of thiols has also some disadvantages. Firstly, they are highly toxic and usually bad smelling, which encumbers their handling; and secondly, the strong coordination of sulfur atom hampers the use of transition metal catalysts. Traditional methods involving electrophilic carbonyl substrates rely on the use of toxic and highly reactive α‐halogenated compounds, that is a pre‐derivatization step leading to stoichiometric amounts of toxic waste.^[18]^ A more straightforward, efficient and atom‐economic way to construct new carbon‐sulfur bonds is the cross‐dehydrogenative coupling (CDC) reaction, which avoids the use of pre‐functionalized substrates. However, the C−H/S−H oxidative coupling between carbonyl compounds and thiophenols is a challenging transformation. In addition to the already commented ability of thiophenols to poison transition metal catalysts, the thioether products could be further oxidized to sulfoxides or sulfones and therefore, the correct choice of the oxidants is crucial. The first two methodologies that allowed the direct oxidative coupling of underivatized carbonyl compounds and thiols, which used CBr_4_ ^[35]^ and *N*‐chlorosuccinimide (NCS)^[36]^ as oxidants were reported in 2008 (Scheme 6). The first one allowed the formation of the corresponding α‐sulfenylated products from a few but very versatile carbonyl substrates (Scheme 6i). Benzenethiol smoothly reacted with acetylacetone, ethyl acetylacetonate, 3‐oxo‐*N*‐4‐chlorophenyl‐ and 2‐methyl‐3‐oxo‐*N*‐phenylbutanamide in the presence of NaOH as the base (71–95 % yield). However only a similar yield was achieved for acetylacetone when 4‐Me‐thiophenol was used. The second methodology does not require the use of a base and it is very operationally simple. With NCS (*N*‐chlorosuccinimide) as the oxidant, a wide variety of cyclic and linear ketones were sulfenylated in good to excellent yields (Scheme 6ii; 21 examples, 72–98 % yield). The atom economy of both procedures is low (37 %) and moderate (61 %) respectively. In contrast, the E factor will be more favorable than for organocatalytic electrophilic sulfenylation procedures. Therefore, the waste generated during these two processes will come mostly from the solvent used during the reaction and the work‐up procedure. The use of oxidants also generates waste, but this affects the E factor less since it represents a minor part of the total waste. However, although it is not highly reflected in the E factor, it should be noted that the oxidants used contain halogens. Furthermore, in both cases the reaction medium is dichloromethane.

**Scheme 7:**
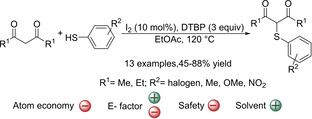
Iodine‐catalyzed oxidative coupling between 1,3‐diketones and thiophenols.

Later, other methodologies based on the oxidative coupling of thiols and underivatized carbonyl compounds have appeared in the literature.[^20a^, ^20b^, ^20e^, ^20f^, ^20h^, ^20i^, ^20j^, ^20k^, ^20l^, ^20m^, ^20n^, ^20o^, ^20t^] For example, 1,3‐diketones have been further studied in the following reports. One of the earliest examples is the iodine‐catalyzed C−H/S−H oxidative coupling of 1,3‐diketones and thiophenols to form β‐dicarbonyl thioethers (Scheme 7).^[20a]^ In this process the oxidant used is di‐tert‐butyl peroxide (DTBP), which was proposed to promote the C−S bond formation through a radical substitution pathway. Although it expanded the number of thioether groups incorporated to acetylacetone, the reaction is very sensitive to the nature of the thiols used. Therefore, despite the electronic nature of the thiols seems to not have an important effect (70–88 % yield), isolated yields were much lower for some thiols, for example in the case of more sterically hindered *o*‐ and *m*‐substituted thiols (45–62 % yield), thiophene‐2‐thiol (28 % yield) or 4‐Me‐ and 4‐F‐thiophenol (51 and 55 % yield, respectively). Furthermore, other substrates with similar structure including 1‐benzolyacetone, beta‐keto ester, dimethyl malonate or even 1,3‐cyclohexanedione are all incompatible with these reaction conditions. Another drawback is that the reaction needs to be performed at high temperature (120 °C) in order to afford the desired products in moderate to good yields (typically 45–88 % yield). This makes the reaction potentially dangerous (the solvent used is ethyl acetate with a boiling point of 80 °C) and therefore reduces the practicality of the process and requires the use of non‐standard equipment. In addition, the need to use the carbonyl substrate in excess and the use of large amounts of oxidants gives a low atom economy (33 %). The E factor is estimated to be quite good since all reagents are commercially available and no further synthetic steps other than the sulfenylation reaction is required. In addition, the reaction is performed in EtOAc and the same solvent is used to extract the product. This opens up the possibility of recycling the EtOAc, thus making the E factor of the whole process favorable. However, the yield is low in some cases, which lowers the E factor.

**Scheme 8:**
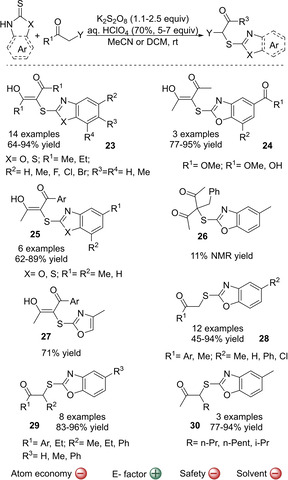
Cross‐dehydrogenative coupling of β‐diketones and monoketones and thiones as sulfenylation reagents mediated by K_2_S_2_O_8_/HClO_4._

Prabhu and co‐workers have contributed to the sulfenylation of both, 1,3‐diketones and monoketones. Their methodology uses thiones that allow the incorporation of several heteroaromatic sulfenyl groups, which are present in many biologically active compounds,^[37]^ to a variety of carbonyl compounds. It should be noted that the introduction of heteroaromatic sulfenyl groups, are usually restricted when using electrophilic sulfur reagents. Moreover, the thiones used are stable crystalline solids and odorless, which facilitate their manipulation as compared with common thiols. The first developed methodology consisted in the cross‐dehydrogenative coupling (CDC) of β‐diketones using K_2_S_2_O_8_ as oxidant in the presence of HClO_4_ (Scheme 8).^[20b]^ The same methodology could also be applied to monoketones.^[20e]^ In both cases a variety of aromatic thiones were used as thiol equivalents to yield the α‐sulfenylated products with good to high yields (up to 96 % yield). Both, symmetrical and unsymmetrical β‐diketones could be used (product **23**–**25**) but not α‐substituted β‐diketone giving product **26**, which showed a very low reactivity (17 % NMR yield). All afforded diketone products were isolated as ketone‐enol tautomers but the authors showed that substrates bearing bulky *tert*‐butyl and phenyl substituents gave the corresponding sulfenylated products predominantly existing in their keto form.^[20b]^ The reaction also tolerated a wide scope of monoketones (products **28**–**30**), although when pure aliphatic ketones bearing a long alkyl chain were used, there was a competition for sulfenylation to both alkyl chains (product **30**). It should be noted that high chemical yields were obtained in the reaction of diketones with both, benzoxazole‐2‐ and benzothiazole‐2‐thiones, but only benzoxazole‐2‐thiones showed reactivity when reacted with monoketones.^[20e]^ This reaction has a low atom economy of 15 % and 22 % for di‐ and mono‐ketones, respectively, owing to the use of the oxidant and a Brønsted acid. In addition, ketones are used in large excess compared to thiones, especially in the case of monoketones (3–4 equivalents), which further decreases the atom economy of the reaction. Nevertheless, the authors showed that the unreacted monoketones can be recovered in the purification process through column chromatography.^[20e]^ Moreover, the E factor of this process will be good, since all reagents are available.

**Scheme 9:**
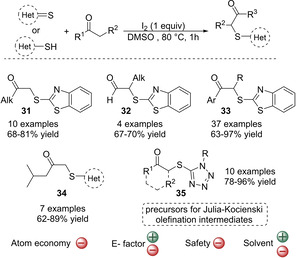
Cross‐dehydrogenative coupling between monoketones and thiones or thiols mediated by I_2._

The same authors reported an alternative methodology that allowed a regioselective sulfenylation of aliphatic ketones by using 1 equivalent of inexpensive iodine as oxidant (Scheme 9).^[20f]^ The α‐CH_3_ group of a range of aliphatic ketones could be sulfenylated selectively in the presence of more reactive α‐CH_2_ or α‐CH groups. Thus, a variety of aliphatic as well as aryl ketones were sulfenylated with benzo[d]thiazole‐2(3H)‐thione showing good to high yields (Scheme 9, 63–97 %). Aldehydes also exhibited good selectivity forming the corresponding α‐sulfenylated products (67–70 % yield), which is remarkable since the aldehyde functionality is prone to oxidation. In this case, not only benzo[d]thiazole‐2(3H)‐thione but also other heteroaromatic thiones could be used. In addition, a variety of heteroaromatic thiols that are commercially available could be efficiently used (Scheme 9, 62–96 % yield). When using thiols, is some cases HI performed better than I_2_. The utility of the methodology was demonstrated by synthesizing precursors for Julia‐Kocienski olefination intermediates (**35**). This procedure allowed to increase the scope of ketones, to give the regioselective sulfenylation of the α‐CH_3_ group when active α‐CH_2_ or α‐CH groups are present in the substrate. It also expanded the scope of thiols/thiones. In contrast to the previous procedure, this method uses dimethyl sulfoxide (DMSO). Although preferred over halogenated solvents such as DCM (dichloromethane) due to the former is cheaper and low‐toxic,^[38]^ DMSO has been listed by several solvent selection guides^[27]^ among the hazardous dipolar aprotic solvents that should be substituted with greener ones. The reason why DMSO is included in that list is not because of its toxicity or environmental impact but mainly because of waste management issues.^[27g]^ The use of DMSO requires large amounts of organic solvents to extract the product, which increases the E factor of the process. Moreover, DMSO can produce an explosion when exposed to halogen compounds such as I_2_ or HI, which hampers the large‐scale applicability of the reaction.^[39]^ Finally, the atom economy is 33 % and 26 % when using thiones and thiols respectively, which is low due to the use of a stoichiometric amount of iodine and again, a large excess of the ketones.

**Scheme 10:**
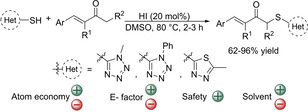
Cross‐dehydrogenative coupling of heteroaromatic thiols and α,β‐unsaturated enones catalyzed by HI.

More recently, the CDC strategy has been expanded to α,β‐unsaturated enones.^[20m]^ In this methodology, a substoichiometric amount of aqueous HI as an additive and DMSO as an oxidant were employed (Scheme 10). The reaction exhibits a high regioselectivity towards sulfenylation of α′‐CH_3_ or α′‐CH_2_ without undergoing conjugate addition, which is difficult to achieve under the CDC method. Good to high yields were obtained for a variety of α,β‐unsaturated arylbutenones and 1‐methyl‐1H‐tetrazole‐5‐thiol (66–96 %). However, the presence of an aromatic group at the β‐position of the enone was necessary so 4‐methylpent‐3‐en‐2‐one and but‐3‐en‐2‐one showed low (32 % yield) or no reactivity, respectively. 5‐Methyl‐1,3,4‐thiadiazole‐2‐thiol was also well tolerated showing good yields (62–78 %). In this process, DMSO was confirmed to act as oxidant and it might be key to promote the regioselective α‐sulfenylation over conjugate addition. The double role of DMSO as oxidant and solvent, reduces the number of reagents used and the amount of HI, which could then be used in substoichiometric amounts. Therefore, the atom economy is improved with up to 64 %, although still a large excess of the substrate ketones is required. However, as described above the use of DMSO affects the E factor of the reaction negatively. Nevertheless, since in this process all reagents used are available, the overall E factor is estimated to be moderately good.

It should be highlighted that all processes reported by Prabhu *et. al*. are operationally simple procedures that do not require the use of sophisticated techniques to work under an inert atmosphere. In addition, this strategy allows the preparation of a range of valuable α‐sulfenylated compounds in a simple and straightforward manner, including β‐diketones, monoketones, α,β‐unsaturated ketones and aldehydes.[^20b^, ^20e^, ^20f^, ^20j^, ^20m^]

**Scheme 11:**
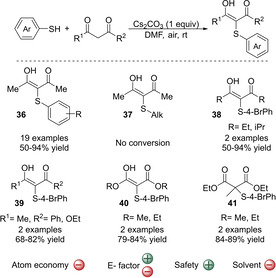
Cs_2_CO_3_‐promoted cross‐dehydrogenative coupling of aromatic thiols and various 1,3‐dicarbonyl compounds catalyzed using O_2_ as oxidant.

Although all these methodologies represent an advancement compared to the traditional methods that use pre‐functionalized substrates, most of the methodologies still have room for improvement since halogens or strong oxidants are required. Since stoichiometric amounts of these reagents are used, the atom economy of the reaction is usually lower than desired. Oxygen (O_2_) has recently emerged as a greener oxidant. However, it is important to distinguish the use of air from the use of pure oxygen. While the former is safe, reactions performed under pure oxygen together with flammable solvents poses a safety risk. Therefore, when implementing protocols that use pure O_2_ in large scales, it is important to apply diligent safety precautions.^[40]^ Nevertheless, procedures employing air or O_2_ are desired against harsh oxidants since they are much safer and benign. In addition, water is formed as a byproduct. In this respect, the Cs_2_CO_3_‐promoted CDC coupling of thiols with β‐diketones under aerobic conditions has recently been reported (Scheme 11).^[20l]^ The reaction is performed in air and therefore, the use of stronger oxidants is avoided. Halogen‐based reagents are not required and only 1 equivalent of a non‐toxic inorganic base (Cs_2_CO_3_) is used. Moreover, the reaction is performed at room temperature under air atmosphere. Therefore, this transformation provides a mild and straightforward route to α‐sulfenylated carbonyl compounds. A wide range of aromatic thiols are well tolerated exhibiting yields up to 95 %, although aliphatic thiols showed no reactivity. Reactions with both symmetrical and unsymmetrical 1,3‐diketones, ‐ketoesters and ‐diesters gave the products **39**–**41** in high yields. This strategy offers a better alternative compared to the CDC couplings described above. It benefits from the advantage of using readily available starting materials and being operationally simple, where a safe and cheap oxidant is used and thereby avoids the generation of large amounts of toxic chemical waste. In addition, it allows the straightforward preparation of a range of α‐sulfenylated carbonyl compounds using safe and available reagents. However, despite all good features of this method there some points to address. First, the atom economy of the reaction is only 36 %, mostly due to the use of stoichiometric amounts of a base and the use of an excess of the thiols. Secondly, the solvent used is DMF (dimethylformamide), which is among the polar aprotic solvents that should be replaced due to its toxicity and environmental impact.^[27g]^ Also as previously commented for DMSO, the use of this class of solvents implies the requirement of large amounts of solvent during the work‐up, increasing the E factor. Therefore, replacement of the solvent by other environmentally friendly solvents would be desirable. The estimated E factor of the whole process is favorable due to the availability of all reagents. Regarding the atom economy, since only H_2_CO_3_ is the only by‐product generated during the whole process, it could perhaps be improved if H_2_CO_3_ is neutralized and recovered for further use. In overall, if both drawbacks commented are solved, this procedure would be a very interesting alternative to prepare α‐sulfenylated carbonyl compounds through the CDC strategy, owing to its simplicity and safeness of the reagents used.

**Scheme 12:**
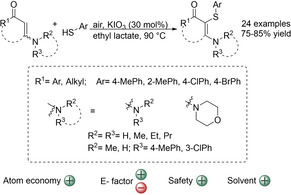
KIO_3_‐Catalyzed aerobic cross‐coupling reactions of enaminones and thiophenols.

Aminothioalkenes are interesting chemical entities that can be converted to many other useful organic compounds.^[41]^ Therefore, various synthetic strategies have been developed to allow efficient preparation of these sulfur‐containing molecules.[^20g^, ^20h^, ^20i^, ^20j^] Among them the KIO_3_‐catalyzed aerobic cross‐coupling reaction of enaminones and thiophenols is noteworthy (Scheme 12).^[20h]^ The catalytic methodology has been used to synthesize a range of aminothioalkene with high yields. Both aryl and alkyl ketone‐based enaminones as well as cyclic ones showed good reactivity. The system also tolerates different substitution patterns on the *N*‐group of the enaminones and the aryl group of the thiophenols. Although most of the enaminones used had (*E*)‐configuration, acyclic enaminones with (*Z*)‐configuration provided similar high yields. Control experiments suggested that KIO_3_ acts as a hypervalent iodate catalyst. The reaction is performed under aerobic atmosphere instead of using harsh oxidants. In addition, it uses simple KIO_3_ as catalyst that avoids the use of large amounts of reagents. Both features translate to an excellent atom economy of 92 %. The starting enaminones can be easily prepared from available diketones and amines, which results in an extra purification step besides the required one to yield the pure aminothioalkenes. Therefore, the E‐factor of this process will be moderate, which could be further improved if recrystallization of enaminones is performed in the cases that it is possible. Another feature of this process is that it employs green bio‐based ethyl lactate (EL) as an efficient solvent. EL is formed by the esterification reaction of ethanol and lactic acid, which can be generated from biomass raw materials through fermentation. Besides being renewable, EL present several advantages such as its low toxicity, easy recyclability and that it is biodegradable and non‐ozone depleting.^[42]^ The only drawback of EL is that its separation from the product might be difficult, as a result of the high boiling point.^[27h]^ Finally, the reaction needs to be heated to 90 °C, which implies a high energy consumption. Nevertheless, this transformation is a promising way to obtain synthetically relevant aminothioalkenes and further studies on it, such as a deeper catalyst screening, might lead to more efficient processes.

**Scheme 13:**
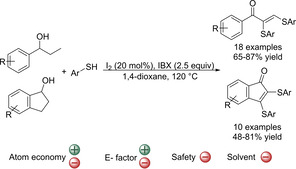
Metal‐free domino synthesis of α,β‐diphenylthioenones from easily available benzylic secondary alcohols and thiophenols employing iodine and IBX.

Very recently it has been shown that readily available benzylic secondary alcohols can also be used for the synthesis of α‐sulfenylated compounds through oxidative couplings. In this respect, a metal‐free domino synthesis of α,β‐diphenylthio enones mediated by iodine and 2‐Iodoxybenzoic acid (IBX) has been reported (Scheme 13).^[20s]^ UV/Vis spectroscopy suggested that a thiophenol‐stabilized iodonium ion is formed in situ that catalyzes multiple steps of the process in a domino manner. The reaction is proposed to proceed via the oxidation of alcohol to ketone, α‐thiolation of ketones followed by domino α,β‐unsaturation, and β‐thiolation of α‐thiolated unsaturated ketones to generate bis‐vinyl sulfides. The reaction has a broad substrate scope for benzylic alcohols (18 examples, 65–87 % yield) and 1‐indanols (10 examples, 48–81 % yield) but acyclic aliphatic secondary alcohols failed to provide the corresponding product. Several aromatic thiophenols could also be used independently of their electronic nature. The reaction thus shows a large substrate scope and it offers an innovative way to obtain α,β‐diphenylthio enones from alcohols, which are readily available in nature. Also, in contrast to most of the utilized oxidants, IBX is nontoxic. The reaction media used is 1,4‐dioxane and the crude product is then extracted with EtOAc. The mixing of both solvents hampers the reuse of the EtOAc employed during the work‐up and thus, it increases the E factor. Nevertheless, the E factor will remain moderately good since the reaction uses readily available materials, so no additional synthetic and purification steps, other than for the obtained pure products, are required. However, 1,4‐dioxane has been catalogued as a solvent presenting some major issues, especially regarding human health and waste management.[^27g^, ^27h^] Therefore, to increase the greenness of the reaction the replacement of 1,4‐dioxane with, for example, 1‐methyl tetrahydrofuran should be considered. To do that, it must be taken into account that IBX has poor solubility.^[43]^ However, it has been shown that at elevated temperatures, IBX is sufficiently soluble in most organic solvents to permit clean oxidations of alcohols.^[44]^ Due to the use of large amounts of oxidants, the atom economy of the reaction is low (31 %). Finally, the boiling point of 1,4‐dioxane is lower than the reaction temperature, so proper reaction vessels for high‐pressure are required.

### 2.3 Oxidative couplings

**Scheme 14:**
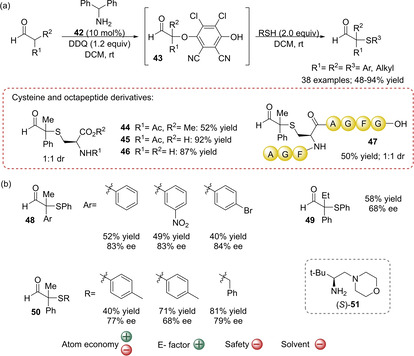
(a) Oxidative coupling of various aldehydes with thiols catalyzed by aminocatalyst **42** and using DDQ as oxidant. (b) Asymmetric version of the coupling of aldehydes with thiols using chiral aminocatalyst (*S*)‐**51**, chloranil as oxidant, and benzoic acid as additive.

Very recently, Jørgensen and co‐workers disclosed the first organocatalytic oxidative α‐thiolation of aldehydes. Instead of an enamine‐electrophile coupling, the authors describe a novel umpolung strategy that allows the activation of aldehydes which can be then paired with different types of nucleophiles, including thiols.^[20r]^ To achieve that, the generated enamine is activated through organocatalysis and quinone‐promoted oxidation to access O‐bound quinol‐intermediates **43** that undergo nucleophilic substitution reactions (Scheme 14). Therefore, the reaction between aldehydes and thiols is in this case allowed, improving considerably the E factor since the preparation of electrophilic sulfur reagents is not required. Another advantage over traditional organocatalytic methods, in which the thioether moiety is limited to the nature of electrophilic sulfur reagents, is that it allows the introduction of several thioether groups. Indeed, several aliphatic thiols, including sterically demanding alkyl groups such as adamantyl, and aromatic thiols with several substitution patterns could be used showing moderate to high yields (66–90 % yield, Scheme 14). Biologically relevant thiols, such as cysteine derivatives, could also be employed maintaining a good catalytic performance (products **44**–**46**, Scheme 14) and this allowed to afford bioconjugates, for example α‐sulfenylated product **47** bearing an octapeptide group (Scheme 14). The reaction also tolerates a large variety of aldehydes, including both aliphatic and aromatic, as well as branched and linear aldehydes (48–94 % yield). Importantly, the authors demonstrated that by using chiral aminocatalyst (*S*)‐**51**, chloranil as the oxidant and benzoic acid as additive, the strategy can also be used to drive the organocatalytic enantioselective coupling of α‐branched aldehydes with thiols (Scheme 14). Chiral quaternary thioethers **48**–**50** were afforded with promisingly good enantioselectivities (66–84 % *ee*) and with moderate to good yields (40–81 % yield). The methodology thus offers an interesting synthetic strategy to afford aldehydes with a quaternary α‐stereocenter from readily available starting materials. This is important since only very few enantioselective nucleophilic substitutions at quaternary stereocenters have been reported, which makes this the first example of a one‐pot enantioselective α‐thiolation of racemic carbonyl compounds. However, the use of DCM as solvent and stoichiometric amounts of 2,3‐dichloro‐5,6‐dicyano‐1,4‐benzoquinone (DDQ) as oxidant affects the greenness of the reaction negatively. In particular, the use of 1.2 equivalents of DDQ affects the atom economy of the reaction negatively, which together with the use of an excess of thiols (2 equivalents) result in a moderate atom economy (46 %). In addition, DDQ is toxic and expensive. Because of the drawbacks of DDQ, numerous reports have appeared in recent years that limits its use in catalytic amounts in combination with stoichiometric milder oxidants,^[45]^ which could be a way to improve the greenness of this very interesting strategy. It should be noted that although the E factor of this process will be moderate due to the availability of all reagents, in the asymmetric version, it will increase since catalyst (*S*)‐**51** is prepared in four steps from readily available L‐*tert*‐Leucine, in which two columns chromatography are performed.

**Scheme 15:**
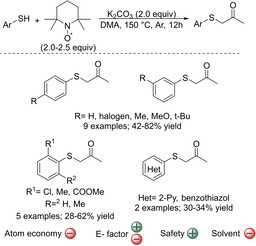
Synthesis of α‐sulfenylated ketones using TEMPO and aromatic thiols through sequential oxidation, skeletal rearrangement, and C−S bond formation.

Besides oxidative couplings between carbonyl compounds and thiophenols, strategies based on other type of substrates, namely amines have also been proposed. Recently, an unprecedented pathway to α‐sulfenylated ketones from commercially available thiols and universally employed TEMPO (2,2,6,6‐tetramethyl‐1‐piperindinyloxy free radical) under simple and metal‐free conditions has been developed (Scheme 15).^[46]^ Besides being the oxidant, TEMPO acts as a C3 synthon through skeletal rearrangement. Mechanistic studies suggest that this reaction involves a consecutive radical oxidation and cation coupling process. During the radical oxidation, TEMPO and thiols serve as oxidants and reductive reagents, respectively. Next, the oxidized thiols are employed as coupling reagents with TEMPO−C3 synthons to yield the corresponding α‐sulfenylated ketones. In general, the yields were moderate to good (42–82 %) with a range of thiophenols with different substitution patterns (Scheme 15). The reaction was quite sensitive to the nature of the thiol and even lower yields were achieved with *ortho*‐substituted thiophenols or heteroaromatic thiols (28–62 %, Scheme 15). In addition, as TEMPO is the source of the C3 synthon, the reaction is limited to obtaining 3‐carbon ketones. In addition to the limited scope, yields are low and TEMPO is used in excess (2.0–2.5 equiv) which increases the E factor of the reaction. The use of TEMPO as C3 synthon makes the atom economy of the reaction low (24 %), since only part of it is incorporated in the final product and the rest becomes chemical waste. Moreover, all reagents are used in excess compared with the thiols. Other drawbacks are that the reaction is performed at high temperature (150 °C) and in dimethylacetamide (DMA), which is toxic. Moreover, despite TEMPO being an inexpensive reagent at a laboratory scale, for industrial applications it would be too expensive. However, the authors showed that the much more economical 4‐Hydroxy‐TEMPO (TEMPOL) could be also used together with thiophenol to give the corresponding ketone in similar yields as with TEMPO (78 vs. 82 %). Therefore, although at this point the methodology presents some features that hampers its applicability, still the authors showed a novel and interesting strategy to afford α‐sulfenylated ketones which is operationally simple and uses available reagents.

**Scheme 16:**
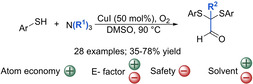
Synthesis of α,α‐disulfenylated aldehydes through oxidative transformation of tertiary amines catalyzed by CuI.

Another example that shows that amines might be considered as a source to prepare α‐sulfenylated carbonyl compounds is the methodology by Pan and co‐workers, in which they used tertiary amines to yield α,α‐disulfenylated aldehydes with oxygen as oxidant and CuI as catalyst (Scheme 16).^[47]^ Interestingly, the initial purpose of the authors was to oxidize a thiophenol derivative in air in the presence of a tertiary amine in order to afford the corresponding sulfinic acid. Instead, a reaction involving a radical pathway took place, which yielded an α,α‐disulfenylated aldehyde. After further optimization of reaction conditions, various aromatic thiols and tertiary amines could be used to afford a range of α,α‐disulfenylated aldehydes in moderate to good yields (35–78 % yield). It should be noted that aliphatic thiols were not reactive under the reaction conditions. As already mentioned, DMSO is not a desired solvent. It should also be noted that after optimization of reaction conditions, pure oxygen was chosen as the oxidant since it gave a slightly higher yield (61 % vs. 72 %). We have already described the danger of using O_2_ in large amounts when operating with flammable solvents and thus, reactions performed under air atmosphere are desired. This issue might be solved by doing some modifications. For instance, performing the reaction in a flow reactor would provide a minimal volume for reaction and a large surface to volume ratio, which would allow the control of the heat generated. Furthermore, this particular reaction could perhaps work in aqueous media, thus minimizing the risk of fire at the same time that DMSO is replaced by a greener solvent. However, chemical waste derived from the tertiary amine will be generated. This affects the atom economy of the reaction negatively, however since the oxidant used is oxygen, both factors will be better than most of the methodologies. In fact, the atom economy is 69 %. Regarding the E factor, the fact that the reagents used are commercially available are favorable compared with other protocols since only a purification step to obtain the pure product is required.

### 2.4 Redox‐neutral couplings

**Scheme 17:**
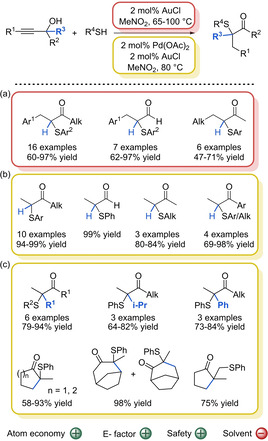
Synthesis of α‐sulfenylated compounds through a hydrothiolation/isomerization process. (a) Scope of the reaction catalyzed by AuCl for primary and secondary propargylic alcohols. (b) Scope of the reaction catalyzed by a mixture of AuCl/Pd(OAc)_2_ for primary and secondary propargylic alcohols. (c) Scope of the hydrothiolation of tertiary propargylic alcohols catalyzed by AuCl followed by semipinacol rearrangement catalyzed by Pd(OAc)_2_ in one pot.

Our group established a completely different approach to prepare α‐sulfenylated compounds in a waste‐free fashion.^[48]^ In 2012 it was found that gold(I) chloride (AuCl) showed an unexpected reactivity when mixing propargylic alcohols and thiols.^[48a]^ Before it had been shown that other metal catalysts, such as rhenium(I), bismuth(III) or iron(III) promote direct substitution of the hydroxyl group to generate the corresponding propargylic thioether.^[49]^ After optimization of the reaction conditions a range of α‐sulfenylated aldehydes and ketones could be obtained in 47–97 % yield (Scheme 17a).^[48b]^ Experimental and DFT (density functional theory) studies showed that the reaction proceeds in two steps. First, regioselective thiolation of the propargylic alcohol forms a sulfenylated allylic alcohol, which isomerizes to the desired α‐sulfenylated ketones.^[48b]^ The methodology allowed the preparation of a wide range of ketones in a mild and efficient way. Indeed, this is a high atom economy route to obtain these valuable building blocks (82 % atom economy). In addition, all reagents are commercially available and therefore, since only the product has to be purified by column chromatography, the overall E factor is not compromised. However, the reaction has some limitations regarding the scope of propargylic alcohols and thiols. If the propargylic alcohols contained a terminal carbon‐carbon triple bond, the regioselectivity and chemoselectivity of the initial gold‐catalyzed hydrothiolation reaction was completely lost resulting in multiple thiol attack to the carbon‐carbon triple bond. Moreover, while primary and secondary aromatic propargylic alcohols could be used, for aliphatic propargylic alcohols only secondary alcohols showed reactivity. Furthermore, only aromatic thiols were found to be reactive under the reported reaction conditions. In this respect, further studies on the system showed that the combination of a Pd/Au catalyst could overcome some of these limitations. The new metal‐mixed system allowed the use of terminal propargylic alcohols as well as both, aromatic and aliphatic alcohols with excellent atom economy (92 %).^[48d]^ Moreover, aromatic groups in the position next to the C−OH moiety were well tolerated (Scheme 17b). Au and Pd/Au systems thus showed a complementary scope. In the latter case, a reaction mechanism was proposed that proceeds through a Pd‐catalyzed regioselective hydrothiolation reaction followed by an Au‐catalyzed 1,2‐hydride migration. The protocol was later extended to tertiary propargylic alcohols, in which a semipinacol rearrangement takes place to produce linear and cyclic tertiary α‐sulfenylated carbonyl compounds (Scheme 17c).^[48e]^ A variety of acyclic alcohols reacted smoothly to generate the products in good to high yields (Scheme 17c; 64–94 % yield), including alcohols with two different substituents at the carbon bearing the hydroxy group. In this case, the migration of the *i*Pr group took place over linear alkyl chains. When an aryl and an alkyl group were present, the aryl displayed a higher migration aptitude than the alkyl group. The use of cyclic propargylic alcohols led to a ring expansion in the final sulfenylated product, maintaining the high yields (Scheme 17c; 58–98 % yield). The utility of the methodology was demonstrated by the D‐ring expansion of an estradiol to obtain novel homo‐D steroids.

**Scheme 18:**
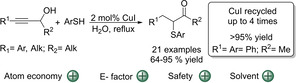
α‐Sulfenylation of propargylic alcohols by aryl thiols in water.

Even though there are many advantages with respect to green chemistry metrics, the use of nitromethane is not desired. In this respect, its replacement by water was studied for the reaction of some propargylic alcohols. Gratifyingly, a CuI catalyst allowed the reaction of various primary and secondary alcohols with an internal alkyne and aromatic thiols in water (Scheme 18).^[48c]^ A range of aldehydes and ketones could be obtained in high yields comparable to the reactions performed in nitromethane (64–94 % yield). Moreover, the catalyst could be recycled four consecutive times without any loss in catalytic activity. After each cycle, the aqueous reaction mixture was extracted using reusable and benign ethyl acetate to remove all traces of the product or reactants. The resulting aqueous solution containing the CuI catalyst could be directly used for the next catalytic cycle. It was also shown that the reaction could be scaled up to 5 grams of propargylic alcohol. It should be noted that this greener protocol is only suitable for primary and secondary alcohols with an internal alkyne and aromatic thiols, thus the substrate scope is rather limited.

**Scheme 19:**
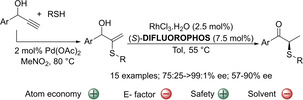
Enantioselective isomerization of allylic alcohols to produce chiral α‐sulfenylated ketones.

Very recently our group showed that this strategy could be used to prepare ketones with a chiral α‐stereogenic center bearing a sulfenyl group.^[48f]^ Prior to that, only two efficient catalytic methodologies were reported for the preparation of chiral α‐sulfenylated ketones.^[50]^ The reason is that organocatalytic α‐sulfenylation of carbonyl compounds are usually restricted to cyclic and/or activated carbonyl compounds such as oxindoles,^[13]^ β‐ketoesters,^[14]^ azalactones,^[15]^ and benzolactones[^14b^, ^14d^] and to a lesser extent to aldehydes.^[16]^ In this respect, a two‐step procedure was developed consisting of a Pd‐catalyzed hydrothiolation of propargylic alcohols followed by an enantioselective Rh‐catalyzed isomerization of the allylic sulfenylated alcohols. The isomerization reaction is the key step for obtaining the ketones in their enantioenriched form, which is challenging since very few procedures exist for this purpose.[^51^, ^52^] Therefore, the focus of this report was on the optimization of this step. After ligand screening, it was found that commercially available (*S*)‐difluorophos ligand in combination with RhCl_3_.H_2_O constituted the best combination to achieve good levels of enantioselectivity. It should be noted that although Rh is an expensive metal, among all Rh‐precursors available in the market, RhCl_3_ ⋅ H_2_O is among the cheapest options. Despite the obtention of good enantioselectivities in most of the cases (typically 87 : 13 to >99 : 1 e.r.), the system is limited to allylic alcohols containing arylthioether groups and aryl ketones (Scheme 19). Control experiments suggested a mechanism involving a Rh‐hydride‐enone intermediate for the isomerization reaction. Overall, it was demonstrated that a 100 % atom economy was achieved in this step. Unfortunately, the two‐reaction process could not be optimized as a one‐pot and this affects the E factor negatively. The solvent used in this case is toluene, which has been catalogued among solvents with some issues, mainly associated with the air impact. In this respect, it would be interesting to replace it by anisole.^[27g]^

## 3 Summary and Outlook

The challenges of handling sulfur‐containing reagents has hampered the development of efficient methodologies towards α‐sulfenylated carbonyl compounds. However, owing to their relevance in synthetic organic chemistry, efforts have been made to find suitable methods for their preparation. These methods rely mostly on the use of electrophilic and nucleophilic carbonyl compounds, including organocatalytic electrophilic sulfenylation, cross‐dehydrogenative couplings, and oxidative couplings. Besides these transformations, redox‐neutral couplings using transition metal catalysis starting from propargylic alcohols have also been reported. The organocatalytic electrophilic sulfenylation, which is usually the choice for preparing chiral α‐sulfenylated carbonyl compounds, gives poor E factors. The reason for this is the requirement of additional synthetic steps for the preparation of electrophilic sulfur reagents. In contrast, oxidative couplings present more favorable E factors, owing to the use of readily available reagents, whereas the atom economy drops since large excesses of oxidants and, in some cases, halogen‐based reagents are required. Moreover, in most of the protocols, the oxidants used are toxic and/or not safe. As an alternative route, redox‐neutral couplings allow the preparation of α‐sulfenylated ketones and aldehydes from available propargylic alcohols and thiols without the use of any harsh oxidant. The green metrics of redox‐neutral protocols are thus improved over electrophilic and oxidant‐based methods. However, they rely on the use of transition metal catalysts that are based on precious metals, whereas other procedures are generally metal free. Moreover, the reaction allows only the preparation of aldehydes and ketones. Another issue that is common in most of the methods developed in academia is the use of solvents that are problematic with respect to human health, the environment, and waste management. This is a concern that we believe should be better addressed by academic research groups. Overall, more research is therefore needed to find suitable methods that allow the preparation of a variety of α‐sulfenylated carbonyl compounds while fulfilling the requirements of green chemistry. Nevertheless, there are already some available protocols that offer promising solutions towards more sustainable methods. For example, tandem reactions, the use of air as oxidant, or the replacement of organic solvents by water have been reported as greener approaches.

The assessment of the greenness of a reaction is not trivial. For a full understanding of the environmental impact of a given synthetic procedure, different metrics need to be considered. For example, it has been shown that several methodologies present high atom economy while the E factor is poor, as well as the opposite situation. Furthermore, these factors do not give information about the safety of a reaction. Therefore, an analysis of all reagents and solvents used must be performed to provide a full overview about the greenness of a reaction. As one reviewer pointed out, it would be beneficial for chemists to have a feasible metric to assess the energy consumption for the synthesis of the desired product. We hope that this Minireview will inspire synthetic chemists to consider atom economy, E factor, and issues regarding safety and solvents when developing new transformations, in addition to developing easily applied metrics to assess energy consumption.

## Conflict of interest

The authors declare no conflict of interest.

## Biographical Information

*Jèssica Margalef received her Ph.D. in 2016 at the University Rovira i Virgili in Tarragona under the supervision of Prof. Montserrat Diéguez and Dr. Oscar Pàmies. During her Ph.D., she did a 3 months exchange in the group of Prof. Hans Adolfsson at Stockholm University and a short spell with Prof. Per‐Ola Norrby at Gothenburg University. In January 2017, she joined Prof. Joseph Samec's group at Stockholm University as a postdoctoral researcher. After two years, she returned to Tarragona as a Martí Franquès postdoctoral fellow. Her research interests include asymmetric homogeneous catalysis, DFT‐guided development of new catalysts, and Ir‐catalyzed water oxidation*.

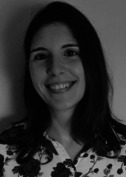

## Biographical Information

*Joseph S. M. Samec received his Ph.D. at Stockholm University in 2005 under the supervision of Prof. J.‐E. Bäckvall and then did a postdoc at California Institute of Technology with Prof. R. H. Grubbs (Nobel Prize Chemistry 2005) as advisor. He then worked as Project Manager for OrganoClick until 2009 when he joined the faculty at Uppsala University. In 2011 he was promoted to Associate Professor. In 2015 he moved to Stockholm University and since 2017, he has been full professor. His research interests concern green and sustainable chemistry with a special emphasis on biomass valorization. In 2013, he founded RenFuel, aimed at valorizing lignin. In 2018 RenCom and LignolProductions and in 2020 RenFuel Materials were founded. These companies are currently at the basic engineering phase to start production of lignin‐based products*.